# Carnosol Alleviates Collagen-Induced Arthritis by Inhibiting Th17-Mediated Immunity and Favoring Suppressive Activity of Regulatory T Cells

**DOI:** 10.1155/2023/1179973

**Published:** 2023-06-28

**Authors:** Jun Chen, Nianzhe Sun, Fuhan Li, Haolin Li, Jiale Tian, Songguo Zheng, Li Zhang, Haidong Wang, Yang Luo

**Affiliations:** ^1^The Department of Neurology, The First Hospital of Lanzhou University, Lanzhou, 730000 Gansu, China; ^2^The First Clinical Medical College, Lanzhou University, Lanzhou, 730000 Gansu, China; ^3^The Second Clinical Medical College, Lanzhou University, Lanzhou, 730000 Gansu, China; ^4^Rheumatic Bone Disease Center, Gansu Provincial Hospital of Traditional Chinese Medicine, Gansu University of Traditional Chinese Medicine, Lanzhou, 730000 Gansu, China; ^5^Department of Clinical Immunology, The Third Affiliated Hospital of Sun Yat-sen University, Guangzhou, China; ^6^Key Laboratory of Biotherapy and Regenerative Medicine, Lanzhou, 730000 Gansu, China

## Abstract

Current approaches are incurable for rheumatoid arthritis (RA). Regulatory T (Treg) cells and T helper cells (Th1 and Th17) are crucial in controlling the process of RA, which is characterized by inflammatory cell infiltration and bone destruction. Carnosol is an orthodiphenolic diterpene that has been extensively applied in traditional medicine for the treatment of multiple autoimmune and inflammatory diseases. Herein, we indicate that administration of carnosol dramatically alleviated the severity of collagen-induced arthritis (CIA) model with a decreased clinical score and inflammation reduction. Cellular mechanistically, carnosol inhibits the Th17 cell differentiation and maintains Treg cell suppressive function in vitro and in vivo. Meanwhile, it also restrains Treg cells from transdifferentiation into Th17 cells under inflammatory milieu. Furthermore, carnosol modulates the function of Th17 and Treg cells possibly via limiting IL-6R (CD126) expression. Collectively, our results suggest that carnosol can alleviate the severity of CIA via hiding Th17 cell differentiation and maintain the stability of Treg cells. Administration of carnosol can be applied as a potential therapy for patients with RA.

## 1. Introduction

Rheumatoid arthritis (RA) is a systemic autoimmune disease characterized by inflammatory cell infiltration and bone destruction [1, 2]. Effector CD4^+^ T (Teff) cells, such as regulatory T (Treg) cells and other T helper cells (Th1 and Th17), are crucial in controlling the process of RA [3, 4]. Importantly, the Th17/Treg imbalance contributes to the pathogenesis and progression of RA [5, 6]. Therefore, targeting Th17 cells or combined with immune suppression can exert a therapeutic effect on RA [7]. Currently, the main treatments for RA are nonsteroidal anti-inflammatory drugs (NSAIDs); however, about 30%-50% of RA patients do not response to the treatment well [8]. Therefore, developing novel candidates is needed to meet the huge demand.

Carnosol is an orthodiphenolic diterpene stemmed from rosemary (*Rosmarinus officinalis*) and mountain desert sage (*Salvia pachyphylla*) [9]. In structure, carnosol has an abietane carbon skeleton with hydroxyl groups at positions C-11 and C-12 and a lactone moiety across the B ring [10]. The special hydroxyl groups endow carnosol with a unique function of antioxidant [10]. Studies confirmed that treatment with carnosol suppressed interferon-*γ-* (IFN-*γ*-) induced inflammatory responses and hydrogen peroxide- (H_2_O_2_-) induced oxidative stress in several types of cancer cells [11]. Carnosol also increases the Nrf2 binding with ho-1-ARE, thereby contributing to HO-1 expression [11]. Moreover, it also promotes intracellular level of glutathione (GSH) and GSH synthesizing enzyme glutamate cysteine ligase catalytic subunit (GCLC) and modifier subunit (GCLM) [10]. Moreover, it also has antioxidant activity. Inflammation induces oxidative stress, resulting in accumulation of reactive oxygen species (ROS), prostaglandins (PGs), cyclooxygenase-2 (COX-2), interleukins (IL), and chemokines, which aggravate tumorigenesis or autoimmune inflammation [12]. Carnosol was verified to attenuate the expression of iCOX-2 and the production of PGE2 in both murine macrophage and human mammary epithelial cells [13].

From the perspective of clinical translation, carnosol has the potential to treat various types of diseases. It has been documented that carnosol was capable in treating LPS-induced septic shock in mice by suppressing inflammasome activation [14]. Additionally, it was effective in treating numerous types of cancers, such as lung cancer [15], colon cancer [16], breast cancer, pancreatic cancer [17], prostate cancer [18], leukemia [19], and brain cancer [20]. Also, carnosol can protect against renal ischemia-reperfusion injury [21] and spinal cord injury [22]. Importantly, carnosol was reported to suppress AIDs, such as ovalbumin-induced allergic asthma [23] and experimental autoimmune encephalomyelitis (EAE) [24]. Li et al. firstly reported that carnosol and rosmanol synergistically alleviate murine arthritis via inhibiting TLR4/NF-*κ*B/MAPK pathway [25]. Given the essential role of Th17 and Treg cell subsets in arthritis, we are wondering whether carnosol can modulate the differentiation and/or function of these cells that contributes to the therapeutic effects on CIA.

In this study, we observed that the administration of carnosol dramatically alleviated the severity of CIA model with a decreased clinic score and joint inflammation. Remarkably, the frequency of Th17 cells was markedly reduced, which was positively correlated with the duration of joint inflammation. Besides, carnosol can also maintain the suppressive capacity of Treg cells in CIA model. In vitro, we confirmed that carnosol inhibits the IL-6R (CD126) expression that can suppress Th17 cell differentiation and promote Treg cell differentiation. Collectively, our results suggest that carnosol can alleviate the severity of CIA via hiding Th17 cell differentiation and maintain the stability of Treg cells; administration of carnosol can be applied as a potential therapy for patients with RA.

## 2. Results

### 2.1. Carnosol Alleviates the Development of CIA by Suppressing Lymphocyte Infiltration

**Figure 1:**
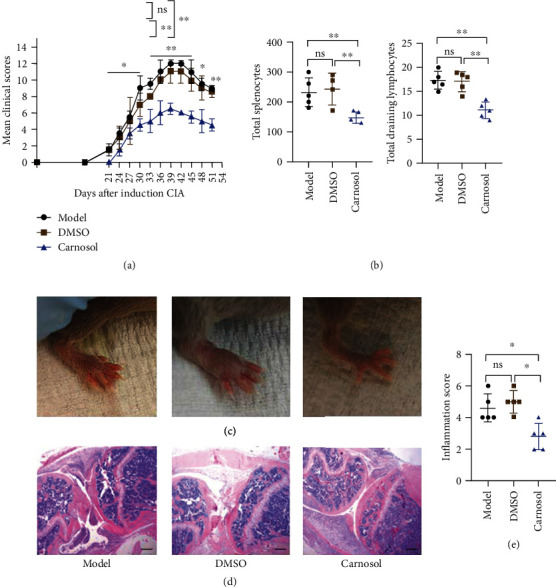
Effects of carnosol on the severity of CIA. DBA1/J mice were immunized with bovine type II collagen in complete Freund's adjuvant and then were injected i.p. with DMSO or carnosol (50 mg/kg) every two days for 14 continuous times (starting on the day of CIA induction). (a) Clinical arthritis scores were determined at various time points after immunization. (b) Numbers of lymphocytes in spleen and dLN from CIA mice. (c) Typical photos (100x). (d) H&E staining results and summary data (e) are shown. Data are presented as mean ± SEM of 5 mice from one of three experiments. ^∗^*p* < 0.05, ^∗∗^*p* < 0.01, and ^∗∗∗^*p* < 0.001 versus DMSO-treated CIA mice group (two-way (a) or one-way (b, d) ANOVA followed by Tukey's multiple comparison test).

To investigate the therapeutic effect of carnosol in the context of autoimmune arthritis, we developed CIA model as previously described [26]. Carnosol was intraperitoneally administrated into the mice every other day after immunization for 14 continuous times at a dose of 50 mg/kg. We observed a significant attenuation in arthritis clinical scores and an obvious delay in CIA onset compared to DMSO administration (*p* < 0.01) (Figure 1(a)). Also, both the lymphocyte numbers in spleen and draining lymph nodes (dLN) were much decreased in carnosol-treated mice, compared with the DMSO group (*p* < 0.01) (Figure 1(b)). Accordingly, joint H&E staining was applied. Histological changes in the whole ankle joints demonstrated a significant decrease in synovitis, pannus formation, and destruction of bone and cartilage after treatment with carnosol (*p* < 0.05) (Figures 1(c) and 1(d)). Together, these results demonstrate that administration of carnosol has a robust therapeutic effect in murine experimental arthritis mice.

### 2.2. Carnosol Reduces the Frequency of Th17 Cells and Local Joint Inflammation in CIA Model

**Figure 2:**
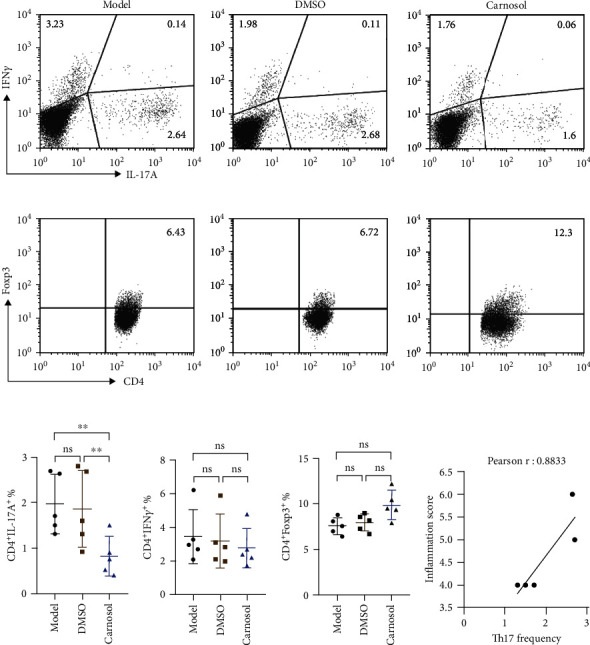
Carnosol decreases the frequency of Th17 cells in CIA model. At day 51 after CII immunization, LN from mice inguinal was harvested, and single-cell suspension was prepared. Populations of T helper cells were analyzed with FACS. (a) Representative flow data showed the frequency of Th1 and Th17 cells from each group. (b) Treg cell frequency from model mice, carnosol, or DMSO-treated mice. Typical FACS plots are shown. (c) Summary data from (a) and (b) are shown. (d) Correlation between Th17 frequency and arthritis scores. Data are presented as mean ± SEM of 5 mice from one of three experiments. ^∗^*p* < 0.05, ^∗∗^*p* < 0.01, and ^∗∗∗^*p* < 0.001 versus DMSO-treated CIA mice group (one-way ANOVA followed by Tukey's multiple comparison test).

As mentioned above, T helper cells are the most important proinflammatory response involved in the pathology of RA/CIA. We hypothesized that carnosol could have an impact on these effector cells. Next, we analyzed the frequencies of Th1, Th17, and Treg cells in carnosol- and DMSO-treated mice individually. As expected, the analysis clearly demonstrated that the number of Th17 cells was markedly declined in the dLN of CIA mice on day 54 compared to model or DMSO group (*p* < 0.01) (Figures 2(a) and 2(c)). Although the frequency of Th1 cells in carnosol treatment group was not statistically significant, it also showed a decreasing trend to some extent (Figures 2(a) and 2(c)). Meanwhile, our results revealed that carnosol was also able to induce Treg responses, which the frequency showed a slight increase, but no difference between model and vehicle group (Figures 2(b) and 2(c)). As is well known, Th17 cells play a driving role in the development of RA by altering the ratio of Treg/Th17 cells [27]. We therefore regressed the correlation between inflammatory scores (Figure 1(d)) and Th17 cell frequency (Figure 2(a)). Consistent with others, we also confirmed that Th17 frequency was positively correlated to inflammatory score in CIA mice (Figure 2(d)). Collectively, these results indicate that carnosol alleviates CIA via suppressing the differentiation of Th17 cells.

Furthermore, we investigated the proinflammatory cytokines and Foxp3 levels in local joints using qRT-PCR analysis. The results revealed that, consistent with the Th17 frequency changing, the mRNA level of IL-17A was significantly decreased in carnosol-treated joint tissues in comparison to model or vehicle group (*p* < 0.05) (Figure S1A, left panel). Although Th1 cells in carnosol-treated CIA mice were not statistically decreased, the mRNA level of IFN-*γ* is lowered with statistical significance in local joint (*p* < 0.05) (Figure S1A, middle panel). However, Foxp3 mRNA in carnosol group was not significantly changed (Figure S1A, right panel). Interestingly, the level of IL-6 mRNA in carnosol-treated CIA joint tissues was significantly decreased (*p* < 0.01) (Figure S1B), implying that carnosol could inhibit joint erosion by suppressing IL-6 secretion. Thus, carnosol not only suppresses the differentiation of Th17 cells but also hides the transcriptional level of proinflammatory cytokines, such as IL-17A, IFN-*γ*, and IL-6, which contribute to the prevention of CIA progress.

### 2.3. Carnosol Stabilizes Treg Cell Suppressive Activity in CIA Model

**Figure 3:**
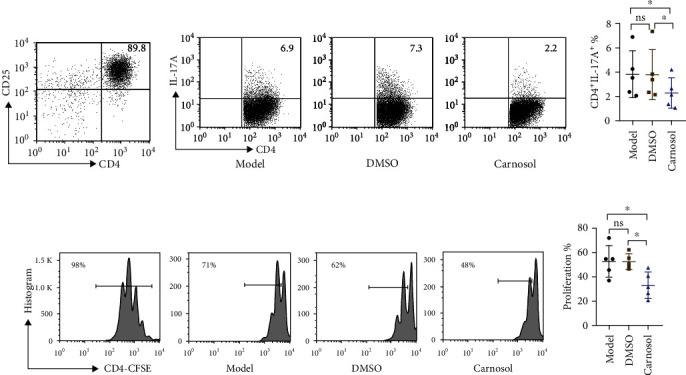
Carnosol stabilizes Treg cells in CIA model. CD4^+^CD25^+^ Treg cells were sorted from arthritic mice on day 51 after CII immunization in carnosol (50 mg/kg), DMSO vehicle alone, and CIA model. Treg cells were then activated with anti-CD3/CD28 in the presence of rmIL-6 for 3 days. (a) The purity of Treg cells was shown. (b, c) The frequency of IL-17A^+^ cells from each group was detected. Typical FACS plots and summary data are shown. (d, e) CFSE-labeled CD4^+^ T cells were cultured with Treg (sorted from model mice) or Treg (sorted from carnosol- or DMSO-treated CIA mice) in the presence of IL-6 (10 ng/mL) at a 2 : 1 ratio for 3 days, and proliferation of cycling CFSE was assessed by flow cytometry. Data are presented as mean ± SEM of 5 mice from one of three experiments. ^∗^*p* < 0.05, ^∗∗^*p* < 0.01, and ^∗∗∗^*p* < 0.001 versus DMSO-treated CIA mice group (one-way ANOVA followed by Tukey's multiple comparison test).

In RA, the stable function of Treg cells plays a pivotal role in maintaining immune microenvironment homeostasis [28]. Therefore, we explored the Treg cell stability in vivo using the established *ex vivo* experiment system. dLN CD4^+^CD25^+^Treg cells were sorted from carnosol- and DMSO-treated CIA mice individually. The purity was confirmed by flow cytometry (Figure 3(a)). As we had expected, CD4^+^CD25^+^IL-17A^+^ Treg cells in carnosol-treated group were statistically lower than those on the corresponding groups after being stimulated with recombinant murine IL-6 (rmIL-6) for 3 days (*p* < 0.05) (Figures 3(b) and 3(c)). These results indicate that carnosol prevents Treg cells from converting to Th17 cells, which may sustain their suppressive function in CIA inflammatory environment. In addition to stability, we also focused on whether carnosol treatment affects the Treg cell immunosuppression. Hence, dLN CD4^+^CD25^+^ Treg cells were harvested from each group on day 54 after CII immunization, and then, a standard in vitro functional assay was performed in the presence of rmIL-6 (Figures 3(d) and 3(e)). The results showed that Treg cells from carnosol-treated CIA mice maintained their suppressive function, which showed a decreased CFSE proliferation, while Treg cells from model-derived or DMSO-treated CIA mice notably lost their suppressive capacity.

### 2.4. Carnosol Diversely Modulates Th17 and Treg Cell Differentiation

**Figure 4:**
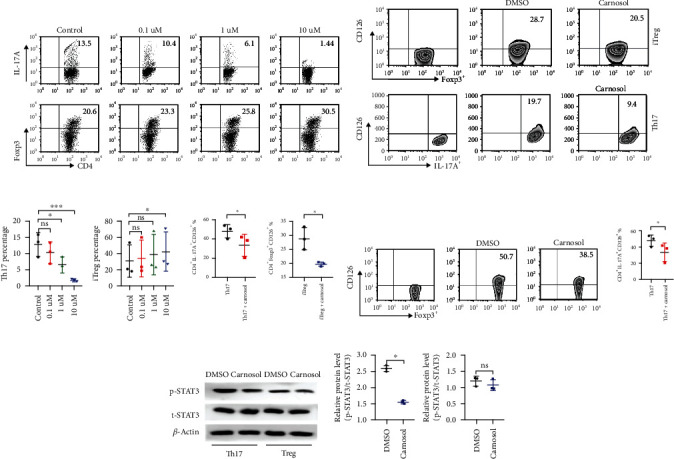
Carnosol restrains Th17 cells and promotes induced Treg cell differentiation possibly via IL-6R signaling. MACS-sorted CD4^+^CD62L^+^ naïve T cells were cultured under Th17- or iTreg-polarizing conditions, in the presence or absence of series concentration of carnosol. (a, c) The frequency of Th17 cells and iTreg cells in treat with carnosol. Typical FACS plots and summary data are shown. (b, d) Representative flow data and statistical analysis of CD126 expression on Th17 and iTreg cell differentiation between carnosol and DMSO treatment. (e, f) CD4^+^CD25^+^ tTreg cells were stimulated with carnosol or DMSO in the presence of IL-6 for 3 days, and CD126 expressions were tested by FACS. (g, h) Naïve CD4^+^ T cells were cultured under Th17-polarizing condition and treated with carnosol (10 *μ*M), or CD4^+^CD25^+^ tTreg cells were stimulated with carnosol (10 *μ*M) in the presence of IL-6 for 3 days. Cells were then analyzed for STAT3 expression by western blot. Data are presented as mean ± SEM of 3 independent experiments. (^∗^*p* < 0.05, ^∗∗^*p* < 0.01, and ^∗∗∗^*p* < 0.001; ns means not significant). One-way ANOVA followed by Tukey's multiple comparison test.

To further confirm the exact function of carnosol on Th17 and induced Treg cell (iTreg) differentiation, we used the established polarizing conditions, respectively. Splenic naïve CD4^+^ T cells were sorted and treated with carnosol at different concentrations. IL-17A or Foxp3 expression was detected by flow cytometry. Consistent with the in vivo results, carnosol significantly suppressed Th17 cell differentiation with a dose-dependent effect (Figures 4(a) and 4(c)). Surprisingly, carnosol can also promote iTreg cell differentiation at the concentration of 10 *μ*M (Figures 4(a) and 4(c)). As we know, Treg cells are heterogeneous and consist of at least two types, tTregs and iTregs [29]. iTregs are resistant to IL-6-driven Th17 cell conversion and maintain the functional capacity in the inflammatory condition [30]. IL-6 -IL-6R (CD126) signaling plays a vital role on CIA/RA progress [31]. Whether carnosol regulates IL-6 signaling in Th17 or iTreg cell differentiation process remains unclear. Given the undetected of IL-6 on T cell subsets, we found that the CD126 expression was quite lower in iTreg and Th17 cells pretreated with carnosol than that pretreated with DMSO control (Figures 4(b) and 4(d)). In addition, we also observed that the tTregs primed with carnosol are also expressed lower CD126 than that on the corresponding cells in the presence of IL-6 (Figures 4(e) and 4(f)). Signal transducer and activator of transcription 3 (STAT3) activities play a crucial role in the differentiation of Th17 cells, which is also one of the key downstream molecule of IL-6 signaling [32]. Therefore, under Th17-polarizing condition, we verified that the phosphorylated STAT3 (p-STAT3) level was significantly decreased in the carnosol-treated group. Similarly, p-STAT3 was also diminished in carnosol-treated tTreg cells (Figures 4(g) and 4(h)). Collectively, these results reveal that carnosol inhibits Th17 cell differentiation with a dose-dependent manner but expedites iTreg differentiation at a relatively high concentration. IL-6R (CD126)-mediated inhibition of STAT3 activity provides a novel explanation for carnosol's role on Th17 and Treg cell differentiation.

## 3. Discussion

Carnosol, the product of oxidative degradation of carnosic acid, has previously been shown to exhibit anti-inflammatory activity and has the potential to treat a variety of inflammatory diseases. Although it has already been shown that carnosol inhibits Th17 cell differentiation and downregulates multiple transcription factors, including NF-*κ*B as well as STAT in EAE model [24], to our knowledge, this is the first study to show that carnosol treatment not only leads to an inhibition in Th17 differentiation but also favors the suppressive activity of Treg cells, contributing to the alleviation of CIA.

The potent arthritogenic effect of Th17 cells mainly lies in the pleiotropic function of IL-17A, which is majorly produced by Th17 cells and acts on the synovial microenvironment: (i) IL-17A acts on the synovial fibroblasts, causing the latter to enhance the production of inflammatory cytokines, such as TNF-*α*, IL-1*β*, and IL-6, (ii) IL-17A recruits macrophages and neutrophils to the site of inflammation in order to cause cell death-mediated inflammation, and (iii) IL-17A accelerates osteoclastogenesis, which leads to bone erosion and cartilage destruction [33]. In addition, IL-22 and IL-21, produced by Th17 cells, alter the glycosylation of autoantibodies and provide them with inflammatory properties. Thus, Th17 cells are potent mediators of arthritis, which coordinate tissue inflammation, cartilage damage, and bone erosion [33]. Mechanistically, IL-6 is crucial to the Th17 cell differentiation. Soluble IL-6 exerts its effects by binding to a receptor complex formed by the ligand-binding IL-6Ra chain (CD126). IL-6 binds to IL-6R, which can then trigger downstream signal transduction and gene expression. The combination of IL-6 and TGF-*β* induces the retinoid-related orphan receptor (ROR) *γ*t, which are the key transcription factors in determining the differentiation of the Th17 lineage [34]. In this study, we showed that carnosol inhibits Th17 differentiation mainly via IL-6-IL-6R signaling.

Although there are many types of Treg, thymus-derived Treg (tTreg) and TGF-*β*-induced Treg (iTreg) are the most predominant Treg cell subsets [28]. tTreg cells are heterogeneous and unstable, which can be converted into other T helper cells in the inflammatory milieu. IL-6 secretion by CIA joints drove the conversion of CD25^+^Foxp3^+^ tTreg cells into pathogenic Th17 cells, and in turn, IL-17A production by the formerly Foxp3^+^ Th17 cells induced more IL-6 from the inflamed synovial fibroblasts, which establishes a positive feedback loop in the arthritic joints [35]. We and others provided more evidence that tTregs were significantly prevented but were less satisfactory in treating autoimmune arthritis because of their plasticity and conversion to Th1, Th17, or T follicular cells under inflammatory conditions [28]. Quite different from the tTreg cells, iTregs are resistant to inflammatory cytokine-driven Th17 cell conversion in the presence of IL-6 and IL-1*β* or even in high salt milieu [28]. Interestingly, we investigated that the effect of carnosol on CIA was also dependent on downregulating inflammatory Th17 cells and enhancing the immunosuppressive function of CD4^+^Foxp3^+^ Treg cells in draining lymph nodes. The frequency of Th17 cell reduction was positively correlated with the degree of arthritis remission. In addition, we also demonstrated that Treg cells from carnosol-treated CIA mice are more resistant to become Th17-like cells, even when stimulated with IL-1*β* and IL-6. Meanwhile, these Treg cells exhibited a markedly enhanced inhibitory activity in suppressing T cell proliferation, compared to the vehicle-treated group. Notably, carnosol can also expedite iTreg differentiation in vitro, indicating that it may exert its protective effects by inducing more stable iTreg cells. IL-6-CD126-STAT3 signaling is essential for the progression of arthritis [32]. Previous findings confirmed that CD126^+^ Treg population is unstable and has compromised immunosuppressive capacity under the inflammatory condition [31]. Herein, we observed that under Th17- or iTreg-polarizing condition, carnosol reduces the CD126 expression on naïve T cells. In other words, carnosol possibly inhibits the initial IL-6R signaling activity, thus leading to lower th17 induction and more differentiation of iTreg cells. One of the downstream molecules of IL6-CD126 signaling is STAT3, which is phosphorylated and that translocate to the cell nucleus, where they act as transcription activators. Finally, we show that carnosol suppressed STAT3 phosphorylation, in response to the ligand IL-6, that blocks Th17 differentiation and favored Treg cell suppressive activity.

In summary, this study demonstrates that carnosol treatment significantly delayed CIA disease onset and decreased the severity of arthritis by downregulating inflammatory Th17 cells while enhancing tTreg cell suppressive capacity. A molecular mechanism of carnosol on CIA arthritis appears to be via an inhibiting effect on Th17 cell differentiation by diminishing STAT3 phosphorylation and favoring Treg cell immunosuppressive function through inhibition of IL-6-IL-6R (CD126) signaling, indicating that administration of carnosol can be applied as a potential therapy for patients with RA.

## 4. Material and Methods

### 4.1. Mice

Male C57BL/6 (B6) and DBA1/J mice were purchased from Lanzhou Veterinary Research Institute. All mice were maintained in a single specific pathogen-free (SPF) room in the animal facility of the Medical College of Lanzhou University. All mice used in this study were 6 to 8 weeks old. All animal experiments were performed with the approval of the Institutional Animal Care and Use Committee of Lanzhou University and according to the approved institutional guidelines and regulations. All protocols were approved by the ethics committee of the First Hospital of Lanzhou University.

### 4.2. Induction and Treatment of CIA

CIA model was developed as previously described [36]. Briefly, bovine type II collagen (CII, 2 mg/ml) was emulsified with complete Freund's adjuvant (CFA) (with heat-denatured mycobacterium 8 mg/ml, Chondrex, LLC, Seattle, WA) at a ratio of 1 : 1. And then, the emulsified mixture was injected intradermally into the tail of DBA1/J mice. Mice were divided into different groups randomly, with or without a single intravenous injection of carnosol (MCE, Cat. No. HY-N0643) or DMSO every other day after immunization for 7 continuous times at a dose of 50 mg/kg. Mice were sacrificed, and data were collected at day 51. The clinical score of arthritis was graded on a scale of 0-4 scales as follows: grade 0, no erythema and no swelling; grade 1, slight erythema and swelling; grade 2, moderate edema and swelling; grade 3, severe swelling and significant edema; and grade 4, severe swelling and significant edema with joint rigidity.

### 4.3. Flow Cytometry

The following anti-mouse monoclonal antibodies conjugated with fluorescein isothiocyanate, phycoerythrin, PerCP-Cy5.5, APC, or Alexa Fluor 647 were purchased from BioLegend (San Diego, CA); CD4 (GK1.5), CD25 (3C7), IFN-*γ* (XMG1.2), IL-17A (TC11-18H10.1), Foxp3 (3G3), and CD126 were from eBioscience. The results were obtained on a BD FACS Calibur flow cytometry and analyzed using FlowJo 10.7. For IFN-*γ* and IL-17A detection, lymphocytes were isolated from spleen, draining LN of CIA mice and stimulated in vitro with PMA (50 ng/ml) and ionomycin (500 ng/ml) for 5 hours, with brefeldin A (10 *μ*g/ml) (all from Calbiochem) added in the last 4 hours, and intracellular expression of IFN-*γ* and IL-17A was analyzed by flow cytometry.

### 4.4. Naïve CD4^+^ T Cell Isolation

Naïve CD4^+^ T cells were prepared as previously described [35]. Single-cell suspensions were obtained from the spleen of C57BL/6 (B6) mice. Splenic erythrocytes were eliminated with red blood cell lysis buffer (Sigma-Aldrich). Total T cells were enriched with nylon wool, and then, naïve CD4^+^ T cells were purified from these T cells via magnetic cell sorting by automagnetic cell sorter (MACS) (Miltenyi Biotec, Germany). In brief, total cells labeled with biotin anti-mouse CD8, CD25, B220, CD11b, CD11C, and CD49b antibodies and anti-biotin microbeads were subjected to a depletion followed by a positive selection with CD62L microbeads by auto MACS. The purity of naïve CD4^+^ T cells was determined as CD4^+^CD25^−^CD62L^+^ with flow cytometry, and the purity of >98% naïve CD4^+^ T cells was used.

### 4.5. In Vitro Differentiation of Mouse T Cells

The protocol for in vitro-polarized Th17 and Treg cells in mice was previously described [37]. Anti-CD3 antibody (BD Biosciences, catalog 553057; 4 *μ*g/ml) in PBS was incubated in 96-well round-bottom tissue culture plates at 4°C overnight. Naïve CD4^+^ T cells were then activated with anti-CD28 (BD Biosciences, catalog 553294; 1 *μ*g/ml): for Th17 induction, TGF-*β* (5 ng/ml, PeproTech), IL-6 (20 ng/ml, PeproTech), anti–IFN-*γ* (10 *μ*g/ml), and anti–IL-4 (10 *μ*g/ml) and for iTreg, TGF-*β* (2 ng/ml) and IL-2 (10 ng/ml, PeproTech). Cells were incubated at 37°C under 5% CO_2_ conditions for 3 to 5 days. The percentages of in vitro-polarized Th17 and Treg cells were analyzed by flow cytometry.

### 4.6. Stimulation of CIA Mice-Isolated Treg Cells

To investigate the stability of Treg cells from in vivo. Using AutoMACS, Treg cells were sorted from carnosol or DMSO-treated CIA mice and untreated model mice individually. The purity of Treg cells was more than 89%. For in vitro stimulation, anti-CD3 antibody (BD Biosciences, catalog 553057; 4 *μ*g/mL) in PBS was incubated in 96-well round-bottom tissue culture plates at 4°C overnight. Treg cells were then activated with anti-CD28 (BD Biosciences, catalog 553294; 1 *μ*g/mL) and stimulated IL-6 (10 ng/ml, PeproTech) for 3 days. For IL-17A detection, cells are stimulated in vitro with PMA (50 ng/ml) and ionomycin (500 ng/ml) for 5 hours, with brefeldin A (10 *μ*g/ml) (all from Calbiochem) added in the last 4 hours, and intracellular expression of IL-17A was analyzed by flow cytometry. CD126 expression was also measured on Treg or Th17 cells after pretreatment with carnosol or DMSO by flow cytometry.

### 4.7. Treg Cell Suppression Assay

MACS-sorted splenic CD4^+^CD25^+^ cells (Treg) from DBA/1J CIA mice (sacrificed on day 51 after CII immunization) were cultured with CFSE (2 *μ*M)-labeled CD4^+^ T cells, with mitomycin C-treated APC (1 : 1), soluble anti-CD3 (1 *μg*/ml), and rmIL-6 (10 ng/ml) for 3 days. Then, cells were harvested, and CFSE proliferation was tested by flow cytometry [38].

### 4.8. Histopathology

For knee joints in CIA, both hind limbs from the CIA mice were dissected and incubated in 10% buffered formalin. Then, the specimens were sectioned and stained with H&E. Two pathologists blindly evaluated the global histological changes according to the infiltration of inflammatory cells and the thickness. For general assessment of histopathology, the paraffin-embedded tissues were sectioned (5 *μ*M), stained with hematoxylin and eosin (H&E), and mounted onto microscope slides for analysis. The histopathologic score was evaluated by a 1–4 scale regarding the degree of cell infiltration, synovial hyperplasia, and cartilage destruction: (1) hyperplasia of the synovial membrane and presence of inflammation infiltration, (2) pannus and cartilage erosion, (3) major erosion of cartilage and subchondral bone, and (4) loss of joint integrity and ankyloses.

### 4.9. Quantitative Real-Time Reverse Transcription PCR

For joint tissue inflammatory cytokine detections, hind limbs from the CIA mice were dissected. Total RNA was extracted using TRIzol RNA Reagent (Invitrogen, Carlsbad, CA, USA) in accordance with the manufacturer's instructions. To determine the relative expressions of cytokines, cDNA was synthesized by equal amounts of RNA from different samples using the PrimeScript™ RT reagent Kit with gDNA Eraser (Takara Bio-technology, Tokyo, Japan) and detected using TB Green™ Premix Ex Taq™ II PCR (Takara Bio-technology). All qPCR reactions were performed on ABI 7500 real-time PCR amplification equipment (Applied Biosystems, Foster City, CA). Primer sequences for PCR were as follows: *β*-actin, forward: 5′-AGCGGT TCC GAT GCC CT-3′, reverse: 5′-AGA GGT CTT TAC GGA TGT CAA CG-3′; IFN-*γ*, forward: 5′-TGA ACG CTA CAC ACT GCA TCT TGG-3′, reverse: 5′-CGA CTC CTT TTC CGC TTC CTG AG-3′; Foxp3, forward: 5′-GGC CCT TCT CCA GGA CAG A-3′, reverse: 5′-GCT GAT CAT GGC TGG GTT GT-3′; interleukin-17A, forward: 5′-AGT GAA GGC AGC AGC GAT CAT-3′, reverse: 5′-CGC CAA GGG AGT TAA AG-3′; and interleukin-6, forward: 5′-AGG AGT GGC TAA GGA CCA AGA CC-3′, reverse: 5′-CTG ACC ACA GTG AGG AAT GTC CAC-3′. The relative expression of target genes was normalized to the internal reference genes GAPDH and U6 and was calculated using the 2^-Ct^ method.

### 4.10. Statistics

Statistical analysis was performed using GraphPad Prism 8(GraphPad Software). Data were analyzed by Student's *t* test in the case of the two groups, and one-way ANOVA analysis was performed for the three groups in mice studies. Data are presented if not indicated elsewhere as mean ± SEM. A value of *p* < 0.05 was considered to be statistically significant (^∗^*p* < 0.05, ^∗∗^*p* < 0.01, and ^∗∗∗^*p* < 0.001; ns means not significant).

## Data Availability

The underlying data related to this paper were made available through contacting Yang Luo (yangluo68@sina.com).

## Supplementary Materials

Supplementary Materials The supplementary information Figure S 1A-1B is available in supplementary material document. Figure S1: qPCR analyses of IL-17A, IFN-*γ*, and Foxp3 in joint tissue samples. Mice were sacrificed at day 51. Total RNA was extracted from each joint tissue sample, and mRNA was quantified by q-PCR; *β*-actin mRNA was used as internal control. The data indicate the mean ± S.E.M (*n* = 10, ^∗^*p* < 0.05, ^∗∗^*p* < 0.01, and ^∗∗∗^*p* < 0.001; ns: not significant).

## References

[1] Smolen J. S., Aletaha D., Barton A. (2018). Rheumatoid arthritis. *Nature Reviews Disease Primers*.

[2] Ahmad S. F., Attia S. M., Zoheir K. M., Ashour A. E., Bakheet S. A. (2014). Attenuation of the progression of adjuvant-induced arthritis by 3-aminobenzamide treatment. *International Immunopharmacology*.

[3] Chen Z., Bozec A., Ramming A., Schett G. (2019). Anti-inflammatory and immune-regulatory cytokines in rheumatoid arthritis. *Nature Reviews Rheumatology*.

[4] Ahmad S. F., Ansari M. A., Nadeem A. (2017). STA-21, a STAT-3 inhibitor, attenuates the development and progression of inflammation in collagen antibody-induced arthritis. *Immunobiology*.

[5] Raychaudhuri S. K., Abria C., Raychaudhuri S. P. (2022). Polyfunctional TEM cells in psoriatic arthritis synovium skewed towards Th17 cells. *Annals of the rheumatic disease*.

[6] Bakheet S. A., Ansari M. A., Nadeem A. (2019). CXCR3 antagonist AMG487 suppresses rheumatoid arthritis pathogenesis and progression by shifting the Th17/Treg cell balance. *Cellular signalling*.

[7] Nygaard G., Firestein G. S. (2020). Restoring synovial homeostasis in rheumatoid arthritis by targeting fibroblast-like synoviocytes. *Nature Reviews Rheumatology*.

[8] Sparks J. A. (2019). Rheumatoid arthritis. *Annals of Internal Medicine*.

[9] Alsamri H., Hasasna H. E., Baby B. (2021). Carnosol is a novel inhibitor of p300 acetyltransferase in breast cancer. *Frontiers in Oncology*.

[10] Kashyap D., Kumar G., Sharma A., Sak K., Tuli H. S., Mukherjee T. K. (2017). Mechanistic insight into carnosol-mediated pharmacological effects: recent trends and advancements. *Life Sciences*.

[11] Satoh T., Trudler D., Oh C. K., Lipton S. A. (2022). Potential therapeutic use of the rosemary diterpene carnosic acid for Alzheimer's disease, Parkinson's disease, and long-COVID through NRF2 activation to counteract the NLRP3 inflammasome. *Antioxidants*.

[12] Karagianni K., Pettas S., Kanata E. (2022). Carnosic acid and carnosol display antioxidant and anti-prion properties in in vitro and cell-free models of prion diseases. *Antioxidants*.

[13] Baradaran Rahimi V., Momeni-Moghaddam M. A., Chini M. G. (2022). Carnosol attenuates LPS-induced inflammation of cardiomyoblasts by inhibiting NF-*κ*B: a mechanistic *in vitro* and *in silico* study. *Evidence-based complementary and alternative medicine*.

[14] Shi W., Xu G., Zhan X. (2020). Carnosol inhibits inflammasome activation by directly targeting HSP90 to treat inflammasome-mediated diseases. *Cell Death & Disease*.

[15] Kalantar H., Sadeghi E., Abolnezhadian F. (2021). Carnosol attenuates bleomycin-induced lung damage via suppressing fibrosis, oxidative stress and inflammation in rats. *Life Sciences*.

[16] Park K. W., Kundu J., Chae I. G., Kim D. H., Yu M. H., Kundu J. K. (2014). Carnosol induces apoptosis through generation of ROS and inactivation of STAT3 signaling in human colon cancer HCT116 cells. *International Journal of Oncology*.

[17] Aliebrahimi S., Kouhsari S. M., Arab S. S., Shadboorestan A., Ostad S. N. (2018). Phytochemicals, withaferin A and carnosol, overcome pancreatic cancer stem cells as c-Met inhibitors. *Biomedicine & Pharmacotherapy*.

[18] Bourhia M., Laasri F. E., Aourik H. (2019). Antioxidant and antiproliferative activities of bioactive compounds contained in *Rosmarinus officinalis* used in the Mediterranean diet. *Evidence-based complementary and alternative medicine*.

[19] Allegra A., Tonacci A., Pioggia G., Musolino C., Gangemi S. (2020). Anticancer activity of Rosmarinus officinalis L.: mechanisms of action and therapeutic potentials. *Nutrients*.

[20] Giacomelli C., Natali L., Trincavelli M. L. (2016). New insights into the anticancer activity of carnosol: p53 reactivation in the U87MG human glioblastoma cell line. *The International Journal of Biochemistry & Cell Biology*.

[21] Zheng Y., Zhang Y., Zheng Y., Zhang N. (2018). Carnosol protects against renal ischemia-reperfusion injury in rats. *Experimental Animals*.

[22] Wang Z. H., Xie Y. X., Zhang J. W. (2016). Carnosol protects against spinal cord injury through Nrf-2 upregulation. *Journal of Receptor and Signal Transduction*.

[23] Lee J. E., Im D. S. (2021). Suppressive effect of carnosol on ovalbumin-induced allergic asthma. *Biomolecules & Therapeutics*.

[24] Li X., Zhao L., Han J. J. (2018). Carnosol modulates Th17 cell differentiation and microglial switch in experimental autoimmune encephalomyelitis. *Frontiers in Immunology*.

[25] Li L., Pan Z., Ning D., Fu Y. (2022). Rosmanol and carnosol synergistically alleviate rheumatoid arthritis through inhibiting TLR4/NF-*κ*B/MAPK pathway. *Molecules*.

[26] Chen J., Huang F., Hou Y. (2021). TGF-*β*-induced CD4+ FoxP3+ regulatory T cell-derived extracellular vesicles modulate Notch1 signaling through miR-449a and prevent collagen-induced arthritis in a murine model. *Cellular & molecular immunology.*.

[27] Liu Y., Jarjour W., Olsen N., Zheng S. G. (2020). Traitor or warrior-Treg cells sneaking into the lesions of psoriatic arthritis. *Clinical Immunology*.

[28] Luo Y., Xue Y., Wang J. (2019). Negligible effect of sodium chloride on the development and function of TGF-*β*- induced CD4^+^ Foxp3^+^ regulatory T cells. *Cell Reports*.

[29] Muñoz-Rojas A. R., Mathis D. (2021). Tissue regulatory T cells: regulatory chameleons. *Nature reviews Immunology*.

[30] Zhang X., Olsen N., Zheng S. G. (2020). The progress and prospect of regulatory T cells in autoimmune diseases. *Journal of Autoimmunity*.

[31] Chen Y., Xu Z., Liang R. (2020). CD4^+^CD126^low/−^ Foxp3^+^ cell population represents a superior subset of regulatory T cells in treating autoimmune diseases. *Molecular Therapy*.

[32] Kishimoto T., Kang S. (2022). IL-6 revisited: from rheumatoid arthritis to CAR T cell therapy and COVID-19. *Annual review of Immunology*.

[33] Hashimoto M. (2017). Th17 in animal models of rheumatoid arthritis. *Journal of Clinical Medicine*.

[34] Huang D. L., He Y. R., Liu Y. J. (2023). The immunomodulation role of Th17 and Treg in renal transplantation. *Frontiers in Immunology*.

[35] Yang S., Zhang X., Chen J. (2020). Induced, but not natural, regulatory T cells retain phenotype and function following exposure to inflamed synovial fibroblasts. *Science Advances*.

[36] Brand D. D., Latham K. A., Rosloniec E. F. (2007). Collagen-induced arthritis. *Nature Protocols*.

[37] Wu R., Zeng J., Yuan J. (2018). MicroRNA-210 overexpression promotes psoriasis-like inflammation by inducing Th1 and Th17 cell differentiation. *The Journal of Clinical Investigation*.

[38] Chen W., Wang J., Xu Z. (2018). Apremilast ameliorates experimental arthritis via suppression of Th1 and Th17 cells and enhancement of CD4+Foxp3+ regulatory T cells differentiation. *Frontiers in Immunology*.

