# Supplementary material for: Carnosol Alleviates Collagen-Induced Arthritis by Inhibiting Th17-Mediated Immunity and Favoring Suppressive Activity of Regulatory T Cells
Source: Biomed Res Int. 2023 Jun 28;2023:1179973. doi: 10.1155/2023/1179973 (PMC10322527; doi:10.1155/2023/1179973)
Supplement: Supplementary Materials — The supplementary information Figure S 1A-1B is available in supplementary material document. Figure S1: qPCR analyses of IL-17A, IFN-γ, and Foxp3 in joint tissue samples. Mice were sacrificed at day 51. Total RNA was extracted from each joint tissue sample, and mRNA was quantified by q-PCR; β-actin mRNA was used as internal control. The data indicate the mean ± S.E.M (n = 10, ∗p < 0.05, ∗∗p < 0.01, and ∗∗∗p < 0.001; ns: not significant). [file 1179973.f1.docx]

**Carnosol alleviates collagen induced arthritis by inhibiting Th17-Mediated Immunity and Favoring Suppressive Activity of Regulatory T-Cells**

**Jun Chen^1#^, Nianzhe Sun^2#^, Fuhan Li^3#^, Haolin Li^4#^, Jiale Tian^2^, Songguo Zheng^5^, Haidong Wang^4*^, Yang Luo^1,6*^**

**^1^** The Department of Neurology, the First Hospital of Lanzhou University, Lanzhou 730000, Gansu, China

^2^ The First Clinical Medical College, Lanzhou University, Lanzhou 730000, Gansu, China

^3^ The Second Clinical Medical College, Lanzhou University, Lanzhou 730000, Gansu, China

^4^ Rheumatic Bone Disease Center, Gansu Hospital of Traditional Chinese Medicine, Gansu University of Traditional Chinese Medicine, Lanzhou 730000, Gansu, China

^5^ Department of Clinical Immunology, The Third Affiliated Hospital of Sun Yat-sen University, Guangzhou, China

^6^ Key Laboratory of Biotherapy and Regenerative Medicine, Lanzhou 730000, Gansu, China

**^#^ These four authors contributed equally to this work.**

***Correspondence and requests for reprints should be addressed to:**

Yang Luo, Address**:** Department of Neurology, The First Affiliated Hospital of Lan Zhou University, Lan Zhou 730000, China; Key Laboratory of Biotherapy and Regenerative Medicine, Lanzhou 730000, Gansu, China; E-mail: [yangluo68@sina.com](mailto:yangluo68@sina.com)

Haidong Wang, Address**:** Rheumatic bone Disease Center, Gansu Hospital of Traditional Chinese Medicine, Lanzhou 730000, Gansu, China; E-mail: [whaidong5895@163.com](mailto:whaidong5895@163.com)


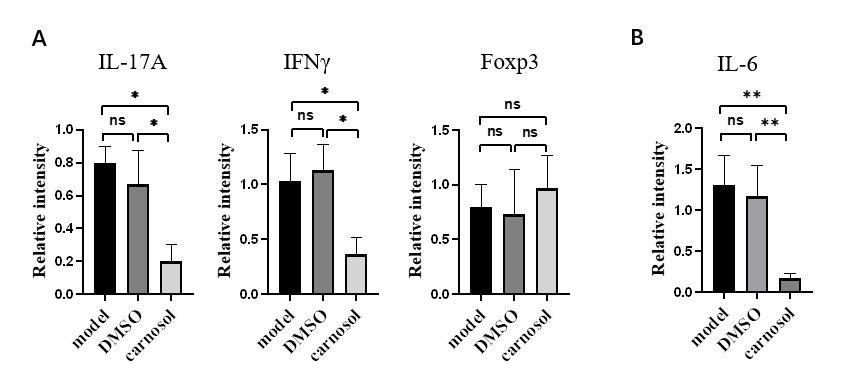


**Figure S1. qPCR analyses of IL-17A, IFN-γ and Foxp3 in joint tissue samples.** Mice were sacrificed at day 51. Total RNA was extracted from each joint tissue sample, and mRNA were quantified by q-PCR, β-actin mRNA was used as internal control. The data indicate the mean ± S.E.M (n=10, *P < 0.05, **P < 0.01, ***P < 0.001, ns, not significant).
